# Case Report: Damaged external limb of montgomery T-tube in a child: a rare complication

**DOI:** 10.3389/fped.2025.1462638

**Published:** 2025-02-21

**Authors:** Ting Wang, Wenjie Wu, Xing Chen, Ling Liu, Huaxu Yin, Bing Xue, Jie Zhang

**Affiliations:** Department of Respiratory and Critical Care Medicine, Chuiyangliu Hospital affiliated with Tsinghua University, Beijing, China

**Keywords:** montgomery T-tube, complication, bronchoscopy, pediatric, airway stenosis

## Abstract

Montgomery T-tubes (MT) are well tolerated with rare complications. Here, we report an unusual complication of damaged external limb of the MT in a child. Both physicians and patients should be aware of this possible complication, which can lead to potential fracture of the MT, resulting in unusual airway problems requiring emergency management.

## Introduction

Montgomery T-tubes (MT) enable patients with laryngotracheal stenosis to maintain airway patency and restore phonation ability. The proximal and distal limbs of the MT are placed into the trachea, whereas the external limb is passed through the artificially created stoma in the front of the neck or previous tracheostomy stoma (1). Generally, MT is well tolerated, and complications are rare. Here, we report another unusual complication: splitting of the external limb of the MT.

## Case report

An 11-year-old boy with progressive dyspnea was admitted to our department. Nine months prior, he suffered from SAVM (spinal arteriovenous malformations) requiring prolonged endotracheal intubation followed by tracheostomy. The patient developed subglottic stenosis because of tracheostomy. He was treated with a pediatric MT (32009R, Boston Medical Products, Inc.) in April 2024. The patients was suggested to inhale acetylcysteine solution(0.3 g, 3 ml) twice a day, and take ambroxol tablets (30 mg) orally three times a day, which enhance mucociliary clearence activities and protect him from mucus retention, and he followed the instructions. A monthly follow-up bronchoscopy was also suggested.

**Figure 1:**
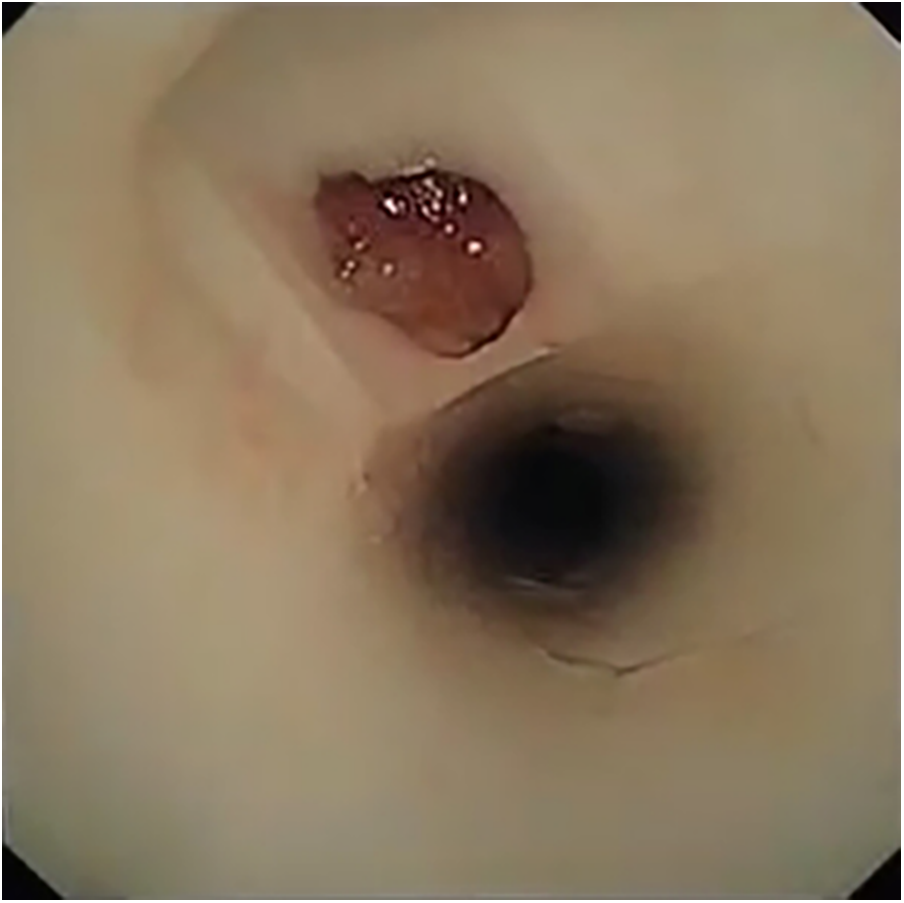
Something soft and round just like sputum bolt is found at the corner between the external limb and the distal limb.

**Figure 2:**
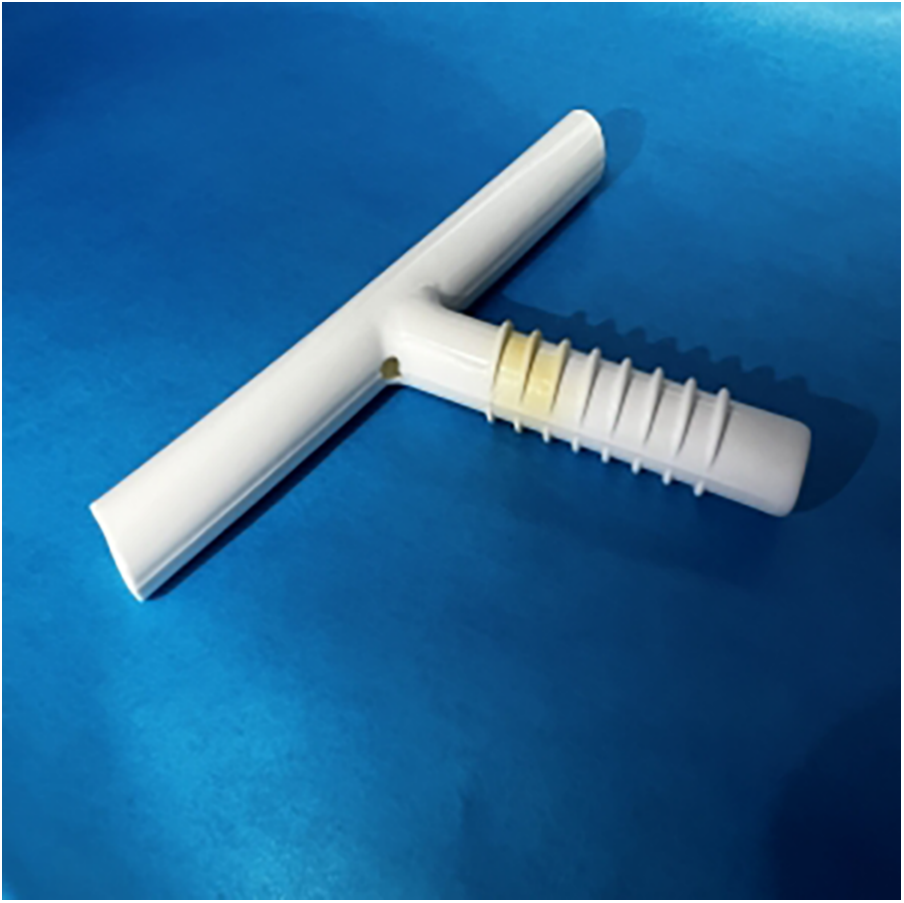
A small hole of approximately 3 × 3 mm was found at the corner.

During the second follow-up bronchoscopy, we found a soft and round object, similar to a sputum bolt, at the corner between the external and distal limbs (Figure 1); however, attempts to remove it by suction proved ineffective. Further efforts to clear it using biopsy forceps failed; finally, we recognized this was the tracheal mucosa. The corner between the external and distal limbs of the MT was damaged. We removed the MT and inserted a new MT to prevent serious fatal complications such as external limb fracture and T-tube migration into the trachea. A small hole of approximately 3 × 3 mm was found (Figure 2).

## Discussion

Granulation tissue formation and mucous plugging are the most common complications of Montgomery T-tubes (2, 3). Migration of the T-tube is exceedingly rare because of its external limb, which works as an anchor to hold it in place (1). However, aspiration of the entire T-tube or parts of the T-tube, such as a plug into the trachea, may occur.

Noirez et al. reported the case of a 44-year-old woman who developed subglottic stenosis because of tracheostomy and was treated with a 12 mm diameter T-tube. However, the external branch of the T-tube suddenly disappeared in the recovery room, necessitating an urgent bronchoscopy. The T-tube was found in the lower trachea. The authors supposed that their common practice of shortening the external branch of the T-tube for esthetic purposes explained the migration (1). S. Srirompotong and K. Yimtae reported another case of T-tube migration (4). The patient coughed vigorously while a nurse cleared secretions from the T-tube, causing sudden aspiration of the external limb of the T-tube through the tracheostomy into the tracheal lumen. The T-tube was then removed using forceps via a rigid bronchoscope. Shinkwin et al. reported a case of plug inhalation (5). A 62-year-old woman with bilateral vocal fold paralysis following thyroidectomy was treated with a T-tube of 14 mm diameter. One day, while replacing the plug, she took a deep inspiration and inhaled it. Subsequently, a rigid bronchoscope was used to remove the plug from the right posterior basal bronchus.

MT is less commonly used in the pediatric age group; therefore, fewer complications were reported in the pediatric age group. A retrospective review performed in 26 children found granulation tissue above the upper limb of the T tube and aspiration to be relatively common complications (6). T. Singh et al. reported fracture of the tracheal limb of MT in a child (7). A 12-year-old boy with subglottic stenosis had a 10 mm external diameter MT. While a surgeon using a curved forceps attempted removal of MT to replace with a tracheostomy tube, the external limb of the MT broke off from the rest of the intratracheal limb. The intratracheal limb of the MT was successfully removed through a tracheostomy.

Herein, we report a case of breakage of the external limb of an MT in a child. Because the external limb of T tube was intact after insertion, we consider the damages to be caused by other postoperative reasons. According to the child's parents' description, they often bent the external limb of the MT forcefully to help the child cough out the sputum (Supplementary Video). Although the breakage mechanism remains unclear, the mechanical stress caused by repeated bending appears to be a likely factor. On the other hand, the rough operation of interventional physicians during operation while stretching the tube to make it pass through the small tracheostomy opening, may be another reason for the damage to the external limb. Finally, compared with adult MT, it seems that the external limbs of pediatric MT are more likely to be broken. We are unsure whether the special angle design of the external limbs in pediatric MT is another factor.

## Conclusion

MT can be used relatively safely, provided that postoperative and home care are meticulous. A broken MT is exceedingly rare; however, physicians should be aware of this complication, which can lead to MT fractures and cause unusual airway problems requiring emergency management. It should be recommended that all patients who received MT insertion undergo regular follow up, and urgent bronchoscopy should be performed if necessary.

## Data Availability

The raw data supporting the conclusions of this article will be made available by the authors, without undue reservation.
